# The inhibitory receptors PD1, Tim3, and A2aR are highly expressed during mesoCAR T cell manufacturing in advanced human epithelial ovarian cancer

**DOI:** 10.1186/s12935-023-02948-0

**Published:** 2023-05-27

**Authors:** Behnia Akbari, Tahereh Soltantoyeh, Zahra Shahosseini, Fariba Yarandi, Jamshid Hadjati, Hamid Reza Mirzaei

**Affiliations:** 1 Department of Medical Immunology, School of Medicine, Tehran University of Medical Sciences, Tehran, Iran; 2 Department of Medical Biotechnology, School of Allied Medical Sciences, Iran University of Medical Sciences, Tehran, Iran; 3 Molecular Virology Department, Pasteur Institute of Iran, Tehran, Iran; 4 Department of Obstetrics and Gynecology, School of Medicine, Tehran University of Medical Sciences, Tehran, Iran; 5 Department of Genetics, University of Pennsylvania Perelman School of Medicine, Philadelphia, PA 19104 USA; 6 Institute for Immunology and Immune Health, University of Pennsylvania Perelman School of Medicine, Philadelphia, PA 19104 USA

**Keywords:** TIM3, A2aR, PD1, Exhaustion, CAR T cell manufacturing, Epithelial ovarian cancer

## Abstract

**Background:**

Chemotherapy and surgery have been the mainstays of epithelial ovarian cancer (EOC) treatment so far. Cellular immunotherapies such as CAR T cell therapy have recently given hope of a cure for solid tumors like EOC. However, extrinsic factors associated with the CAR T cell manufacturing process and/or intrinsic dysregulation of patient-derived T cells, which could be associated with cancer itself, cancer stage, and treatment regimen, may hamper the efficacy of CAR T cell therapy and promote their exhaustion or dysfunction.

**Methods:**

To investigate the association of these factors with CAR T cell exhaustion, the frequency of T and CAR T cells expressing three immune inhibitory receptors (i.e., TIM3, PD1, A2aR) generated from T cells of EOC patients and healthy controls was measured during each stage of CAR T cell production.

**Results:**

Our findings revealed that primary T cells from EOC patients show significantly elevated expression of immune inhibitory receptors, and this increase was more prominent in patients undergoing chemotherapy and those with advanced cancer. In addition, the CAR T cell manufacturing process itself was found to upregulate the expression of these inhibitory receptors and more importantly increase the population of exhausted mesoCAR T cells.

**Conclusions:**

Our observations suggest that intrinsic characteristics of patient-derived T cells and extrinsic factors in CAR T cell production protocols should be considered and properly counteracted during CAR T cell manufacturing process. In addition, mitigating the signaling of immune inhibitory receptors through pharmacological/genetic perturbation during CAR T cell manufacturing might profoundly improve CAR T cells function and their antitumor activity in EOC and other solid tumors.

**Supplementary Information:**

The online version contains supplementary material available at 10.1186/s12935-023-02948-0.

## Introduction

Ovarian cancer is the third most common gynecologic malignancy worldwide, but it has the highest mortality rate among these cancers [1]. Typically, ovarian cancer is diagnosed at a late stage and lacks an effective screening strategy [1, 2]. Epithelial ovarian cancer (EOC) is the most common type of ovarian cancer in women [3]. More than 80% of EOC cases are diagnosed at an advanced stage when the tumor has spread to the peritoneal cavity and upper abdominal organs [3]. EOC metastasis greatly reduces the chance of a cure as five-year survival rate drops significantly with the spread of EOC to other organs and tissues beyond the pelvic cavity [3]. Standard treatments for newly diagnosed EOC usually involve cytoreductive surgery and platinum-based chemotherapy [1–3]. However, EOC cases often relapse and become resistant to chemotherapy [1, 3], highlighting the urgent need for the development of targeted therapies that can specifically target the cancer cells and spare healthy cells, leading to better treatment outcomes and improved quality of life for patients. Targeted therapies can include drugs that target specific molecular pathways or proteins that are overexpressed or mutated in cancer cells, or immunotherapy approaches-such as adoptive T cell-based immunotherapies- that harness the power of the immune system to recognize and destroy cancer cells.

Adoptive T cell-based immunotherapies that utilize genetically engineered T cells modified to express a chimeric antigen receptor (CAR) or cloned T cell receptor (TCR) have shown durable clinical responses in patients with various cancers [4–6]. The success of adoptive cellular immunotherapies has led to regulatory approval of several anti-CD19-targeted CAR T cell therapies [7]. Mesothelin, a 40 kDa protein, is highly expressed in tumor tissues of patients with serous or endometrioid EOC [8, 9]. Data also indicate that advanced tumor stages (III and IV) and high-grade EOC patients express higher levels of mesothelin in their tumors [10, 11]. Anti-mesothelin CAR T cells (mesoCAR T cells) are currently being used in clinical trials for various types of tumors, including EOC (NCT03054298, NCT05057715, NCT04503980, NCT03814447, NCT03916679, NCT02159716). Overall, mesoCAR T cell therapy appears to be a promising treatment option for patients with high-grade EOC. However, the clinical application of mesoCAR T cells has been limited due to intrinsic and extrinsic factors that induce CAR T cell dysfunction and exhaustion [7]. Among these intrinsic factors, the quality of patient-derived peripheral T cells is of paramount importance, as these cells are essential for generating functional autologous CAR T cells [12]. Moreover, during CAR T cell manufacturing, T cell activation and viral transduction are considered extrinsic factors that can impact the anti-tumor potency of CAR T cells [13]. We hypothesize that these intrinsic and/or extrinsic factors may contribute to CAR T cell dysfunction and exhaustion. Therefore, we investigated the effects of intrinsic factors (i.e., patient-derived T cells, cancer stage, and treatment regimen) and extrinsic factors (i.e., T cell activation and viral transduction) on the induction of mesoCAR T cell exhaustion.

## Materials and methods

### Study population

Thirty adult patients who had been clinically and pathologically diagnosed with stage III and IV epithelial ovarian cancer, with an average age of 52.7 were enrolled in the study (Table S1). These patients were admitted to Yas Hospital of Tehran University of Medical Sciences, Iran, and provided their consent under institutional review board-approved research protocols at the National Institute for Medical Research Development of Iran [IR.NIMAD.REC.1399.287]. Thirty age- and sex-matched healthy adults were also involved in the study as healthy controls.

### Sample collection and PBMC isolation

The whole blood was collected into 3 mL K3-EDTA vacutainer tubes (Becton-Dickinson), mixed immediately after collection by inverting 10 times, and shipped to the lab in less than 30 min. Peripheral blood mononuclear cells (PBMCs) were isolated using Histopaque®-1077 (Sigma Aldrich). Briefly, the whole blood was diluted 2-3x with phosphate-buffered saline (PBS). Sixteen mL of the cell suspension was then carefully layered over 8 mL of Histopaque in a 50 mL conical tube. The cells were next centrifuged at 800 g for 20 min with the brake off. The buffy coat was carefully harvested and transferred into a new 15 mL conical tube. The cells were then resuspended in 10 mL of PBS buffer and centrifuged at 500 g for 10 min. To remove platelets, the cell pellet was resuspended in 5 mL of PBS buffer and centrifuged at 100 g for 3 min. The cells were then counted using a hemocytometer with trypan blue to determine viability. Finally, PBMCs were cryopreserved in a cryoprotective medium containing 10% dimethyl sulfoxide (Sigma Aldrich) and 90% fetal bovine serum (Gibco), stored at -80 °C, and transferred to a nitrogen tank for long-term storage.

### Antibodies and cytokines

PE anti-human PD-1 (Cat.no: 329,906, clone EH12.2H7), APC anti-human Tim-3 (Cat.no: 364,804, clone A18087E), and isotype controls (Cat.no: 400,112 and 400,122, clone MOPC-21) were purchased from Biolegend. Alexa Fluor® 647 anti-human Adenosine A2aR (Cat.no: FAB94971R, clone #599,717), and isotype control (Cat.no: IC003R, clone #20,102) were purchased from R&D Systems. For T cell enrichment and stimulation, anti-human CD3 (Cat.no: 130-093-387, clone OKT3) and anti-human CD28 (Cat.no: 130-093-375, clone 15E8) antibodies and Recombinant human IL-2 IS, research grade (Cat.no: 130-097-743) were purchased from Miltenyi Biotec.

### Generation of lentiviral vectors

Second-generation replication-defective lentiviruses were produced using a standard calcium phosphate transfection protocol. A third-generation transfer plasmid encoding GFP, and anti-human mesothelin-BBζ CAR, along with two packaging plasmids encoding VSV-G (pMD2.G) and Gag, Pol, Tat, and Rev (psPAX2), were transfected into Lenti-X 293T cell line using the calcium phosphate transfection method [14]. Viral supernatants were harvested 24- and 48-hours post-transfection and concentrated using high-speed centrifugation at 55,000 g for 2 h at 4 °C. The concentrated lentiviruses containing the transfer plasmid were then titered and stored at -80 °C.

### T cell activation and CAR T cell production

T cells were enriched and activated using soluble anti-hCD3 and anti-hCD28 antibodies at concentrations of 1 µg/mL and 3 µg/mL, respectively. After 4 days, the cells were washed, and the purity of T cells was checked using anti-hCD3 antibodies via flow cytometry. The T cells were resuspended at a concentration of 1 × 10^6^ cells per ml in RPMI 1640 medium (Gibco) supplemented with 10% FBS (Gibco), 2 mM L-glutamine (Sigma Aldrich), 20 mM HEPES (Sigma Aldrich), 8 µg/mL Polybrene (Santacruz), and 100 IU/mL IL-2 (Miltenyi Biotec). The T cells were then transduced with concentrated viral vectors at a multiplicity of infection (MOI) of 10. Spinfection at 850 g for 60 min was performed to enhance transduction efficacy. After 3 h, 2 mL of complete medium was added to the transduced T cells for every 1 × 10^6^ cells. Four to seven days post-transduction, the expression of GFP, PD-1, TIM-3, and A2aR were assessed using flow cytometry.

### Flow cytometry

Cell surface expression of PD-1, TIM-3, and A2aR was measured using flow cytometry in three main stages: after thawing, post-activation, and post-transduction. Briefly, cells were washed with fluorescence-activated cell sorting buffer consisting of PBS, 2% FBS, and 5 mM EDTA. Cell count was determined, and 10^5^ cells were labeled with fluorochrome-conjugated antibodies based on the manufacturer’s instructions. The cells were then incubated with antibodies for 30 min at 4 °C. Cells were subsequently washed and resuspended in FACS buffer. Positively stained cells were distinguished from the background using fluorescence-minus-one controls. Flow cytometry was performed using a BD FACSCalibur flow cytometer and analysis was done using FlowJo software (Tree Star Inc., version 10.6). All statistical analyses were conducted using GraphPad Prism (Version 8.3.0).

## Results

### Patient-derived T cells express higher levels of immune inhibitory receptors compared to healthy T cells

Thirty patients diagnosed with high grade and late stage (III and IV) epithelial ovarian cancer (EOC) were enrolled in this study. Among them, 20 patients were receiving Carboplatin-Taxol (CarboTaxol) as conventional treatment for high grade EOC, while the remaining 10 patients were newly diagnosed and had not yet started treatment. Additionally, 30 age-matched female healthy controls with no history of disease in the past three months were also enrolled in this study.

**Fig. 1:**
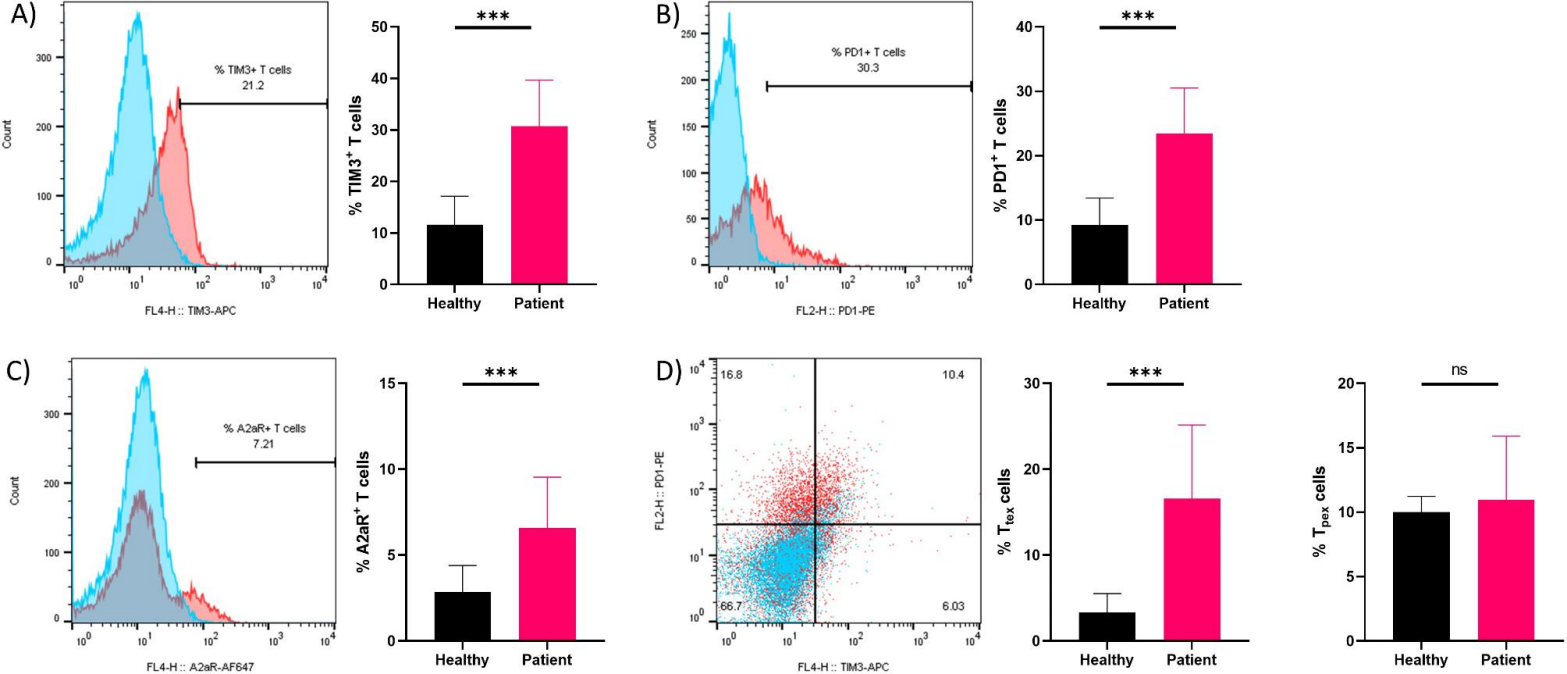
Baseline expression level of inhibitory receptors in T cells of patients with EOC and healthy controls. The baseline percentage of TIM3+ **(A)**, PD1+ **(B)**, and A2aR+ **(C)** cells are significantly higher in EOC patient-derived T cells post thawing. The frequency of T_tex_ cells is significantly higher in patients’ samples compared to normal controls **(D)**. However, the frequency of T_pex_ cells is not significant different between patients and healthy samples (D). Representative overlay histogram plots representing expression level of TIM3 (A), PD1 (B), and A2aR (C) in EOC patient-derived T cells sample (Red color) vs. unstained sample (Blue color). Representative overlay dot plot (D) representing the frequency of terminally exhausted and progenitor exhausted T cells in an EOC patient sample (Red color) vs. unstained sample (Blue color). Data from healthy donors (n = 30) and EOC patients (n = 30) were analyzed using Welch’s t-test (A and B) Mann-Whitney U test (C and D). *P < 0.05; **P < 0.01; ***P < 0.001. Data are presented as mean ± SD.

Previous studies have shown that PBMCs and T cells from cancer patients have inherent metabolic and epigenetic dysregulations [15–17]. To investigate the potential differences in immune inhibitory receptor expression, we measured the baseline expression of PD1, TIM3, and A2aR in patient-derived PBMCs and T cells and compared them with healthy controls (Gating strategy is shown in Figure S1). We observed that PBMCs of EOC patients significantly express higher levels of TIM3 and PD1 compared to healthy PBMCs (Figure S2A, S2B). However, no significant difference was observed for A2aR expression (Figure S2C). Next, to determine if obtained results are restricted to T cells or other mononuclear cells present in the PBMC fraction, we stained our samples with anti-CD3 antibody. Interestingly, we found that patient-derived T cells significantly express higher levels of immune inhibitory receptors (TIM3, PD1, A2aR) compared to healthy control T cells (Fig. 1A C).

Notably, previous studies have categorized exhausted T cells into distinct groups based on their function and phenotype: progenitor exhausted T cells (T_pex_) and terminally exhausted T cells (T_tex_) [18, 19]. Progenitor exhausted T cells that express PD1 but not TIM3, retain polyfunctionality and exhibit stem-like properties. On the other hand, terminally exhausted T cells that express both PD1 and TIM3 at higher levels, are short-lived, and are unable to control tumor growth [20]. While T_pex_ cells can be rejuvenated by immune checkpoint blockade, T_tex_ cells cannot [21]. In line with this notion, our data revealed that T_tex_ cells were present in peripheral T cells of EOC patients, and this fraction was significantly enriched compared to healthy controls (Fig. 1D). However, no significant difference in the population of T_pex_ cells was observed between patients and healthy controls (Fig. 1D). Overall, our data suggest that patient-derived T cells overexpress immune inhibitory receptors and may have potentially reduced immunological function compared to normal T cells.

### CAR T cell manufacturing process promotes the expression of immune inhibitory receptors

**Fig. 2:**
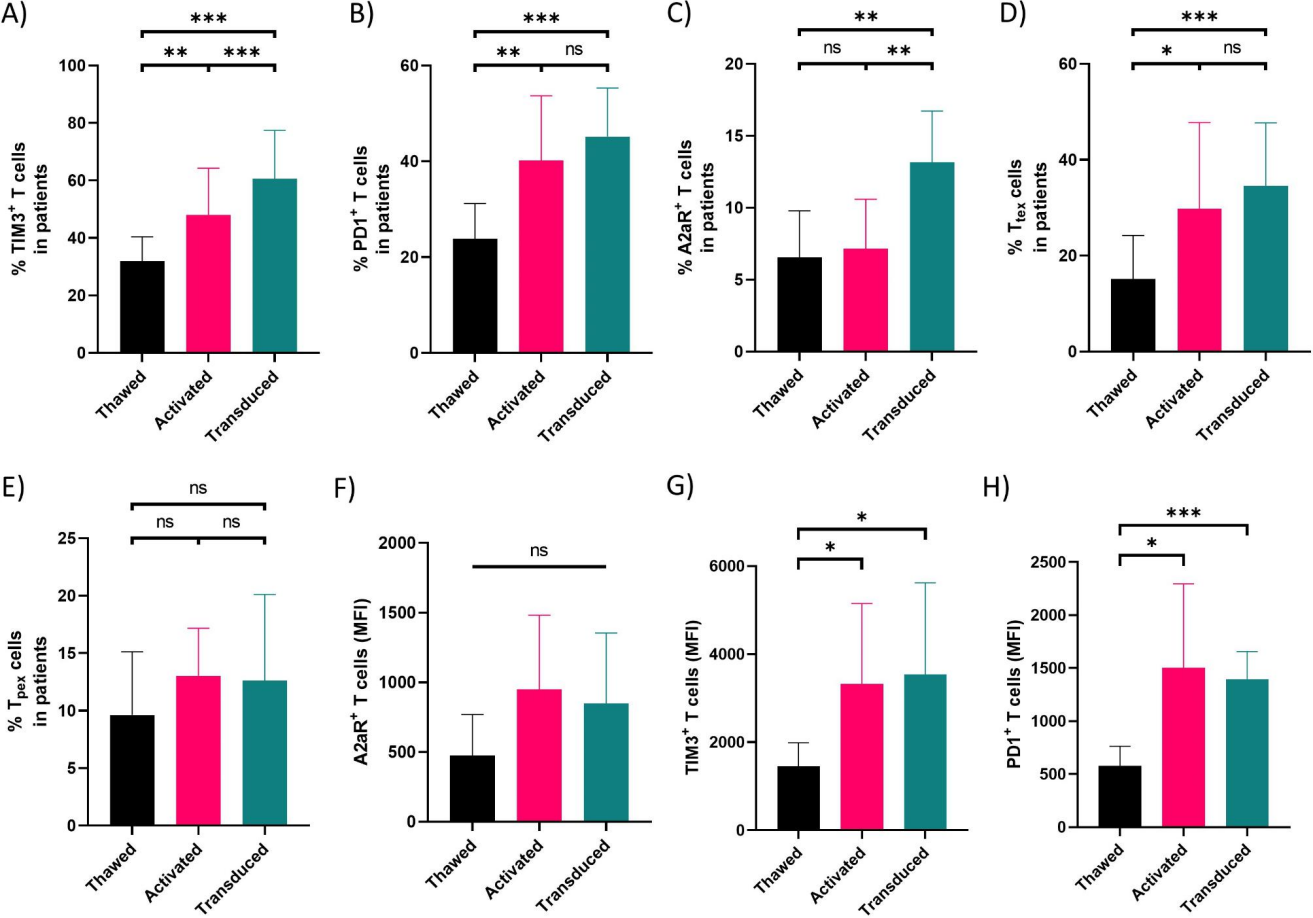
Expression pattern of inhibitory receptors during CAR T manufacturing process. CAR T cell population positive for TIM3 **(A)**, PD1 **(B)**, and A2aR **(C)** in EOC patient-derived T cells during CAR T cell manufacturing steps. The frequency of T_pex_**(D)** and T_tex_**(E)** cells during manufacturing steps of CAR T cells derived from EOC patient T cells. Median fluorescence intensity (MFI) of A2aR **(F)**, TIM3 **(G)**, and PD1 **(H)** in EOC patient-derived T cells during CAR T cells manufacturing process. Healthy donors (n = 30) and EOC patients (n = 30) data were analyzed using RM one-way ANOVA and Tukey multiple comparison test. *P < 0.05; **P < 0.01; ***P < 0.001. Data are presented as mean ± SD.

The process of CAR T cell production begins with the isolation of T cells from the peripheral mononuclear cells (PBMCs) of patients. Isolated T cells are then activated through in vitro engagement of T-cell receptors (TCRs) and costimulatory molecules (i.e., CD28) with their cognate ligands, followed by transduction of activated T cells with a viral vector-encoding CAR transgene, and their ex vivo expansion before re-infusion. Previous studies have shown that rapid ex vivo proliferation of T cells can result in a partial loss of antitumor potency [13]. Therefore, we sought to investigate whether the CAR T cell manufacturing process could alter the immunophenotype of T cells by affecting the expression of immune inhibitory receptors. To address this question, we activated and transduced T cells from healthy controls (Figure S2D). Our results demonstrated a significant enrichment of TIM3 + and PD1 + T cell population during T cell activation in healthy controls (Figures S3A, S3B). However, the frequency of A2aR + T cells did not show a significant change during T cell activation (Figure S3C). Interestingly, the progenitor and terminally exhausted fraction of T cells were also significantly upregulated during T cell activation (Figure S3D, S3E). Subsequently, activated T cells were transduced with 2nd generation mesoCAR vector. Four days post transduction, the percentage of PD1 + T cells, TIM3 + T cells, A2aR + T cells, as well as the percentage of T_tex_ cells were significantly increased (near 2-fold) in healthy controls (Figures S3A-S3C, S3E). We next repeated the experiment with EOC patient-derived T cells. We observed a significant increase in the frequency of PD1 + and TIM3 + cells in both activated T cells and generated mesoCAR T cells compared to freshly isolated T cells from patients (Fig. 2A and B). The A2aR + T cells were only increased significantly in the generated mesoCAR T cells compared to freshly isolated T cells (Fig. 2C). Interestingly, we observed a significant increase in the abundance of TIM3 + as well as A2aR + mesoCAR T cells compared to activated T cells (Fig. 2A and C). Moreover, T_tex_ cells were significantly enriched during mesoCAR T cell manufacturing process in patient’s samples (Fig. 2D). However, no significant change was observed in T_pex_ cells of patient’s samples during T cell activation or viral transduction (Fig. 2E). Consistent with our findings on the enrichment of PD1 + and TIM3 + T cell subset (i.e., T_tex_ cells), median fluorescence intensity (MFI) analysis also revealed a significant upregulation of TIM3 and PD1 expression, but not A2aR expression, on T cells during the CAR T cell manufacturing process (Fig. 2F H). Altogether, it can be concluded that the expression of immune inhibitory receptors PD1, TIM3 and A2a, is increased during CAR T cell manufacturing process and more importantly, it seems that current CAR T cell production protocols can lead to ex vivo differentiation of CAR T cells into terminally exhausted phenotype especially in cancer patients.

### Patient-derived T cells express higher levels of inhibitory receptors during CAR T cell manufacturing process compared to healthy controls

**Fig. 3:**
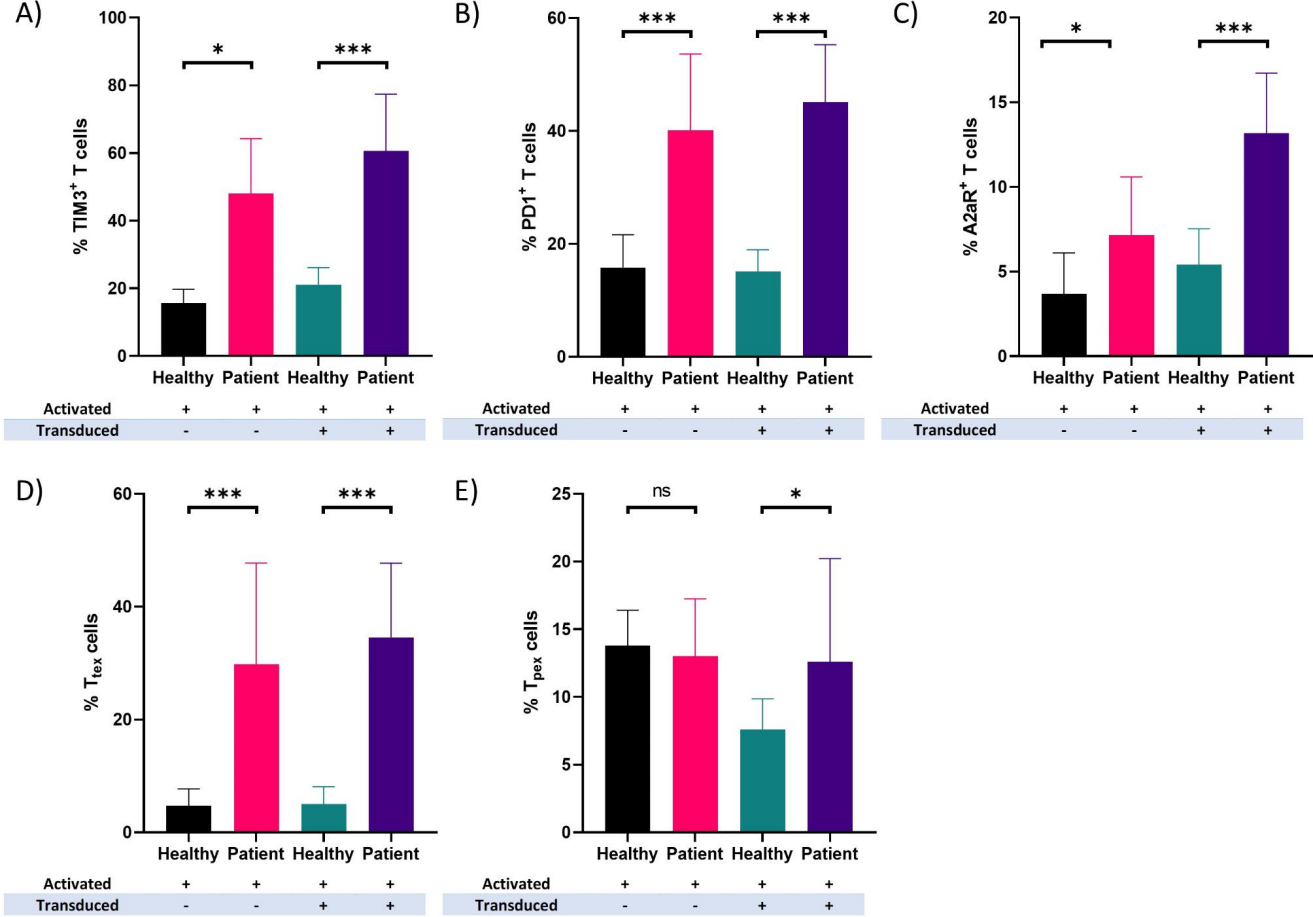
Expression pattern of inhibitory receptors in patient-derived CAR T cells. CAR T cell population positive for TIM3 **(A)**, PD1 **(B)**, and A2aR **(C)** in EOC patient-derived T cells after activation and transduction vs. healthy controls. The frequency of T_tex_**(D)** and T_pex_**(E)** cells after activation and transduction of EOC patient-derived T cells vs. healthy controls. Data from healthy donors (n = 30) and EOC patients (n = 30) were analyzed using Mann-Whitney U test (A, B, and D) and Welch’s t-test (C and E). *P < 0.05; **P < 0.01; ***P < 0.001. Data are represented by mean ± SD.

To further investigate the intrinsic differences between the patient-derived CAR T cells and healthy control-derived CAR T cells, we generated CAR T cells from T cells of healthy donors and EOC patients, and examined the alteration in frequency of TIM3+, PD1+, and A2aR + cells. We have previously observed that the percentage of the T cells positive for these three immune inhibitory receptors are significantly higher in patient-derived T cells compared to normal T cells even prior to T cell activation (Fig. 1A C). Further analysis showed that activated T cells from EOC patients have a significantly higher percentage of potentially scarred T cells (PD1+, TIM3+, or A2aR + T cells) compared to activated T cells from healthy controls (Fig. 3A C). Interestingly, the percentage of T_tex_ cells were profoundly higher in patient-derived activated T cells compared to healthy controls (Fig. 3D). Conversely, the percentage of T_pex_ cells was not significant between two groups upon T cell activation (Fig. 3E). Furthermore, mesoCAR T cells generated from patient-derived activated T cells showed a significant enrichment in population of potentially scarred T cells compared to healthy controls (Fig. 3A C). Additionally, the percentage of both exhausted T cell subsets (i.e., T_tex_ and T_pex_ cells) was significantly higher in patient-derived mesoCAR T cells compared to healthy control-derived mesoCAR T cells (Fig. 3D and E).

**Fig. 4:**
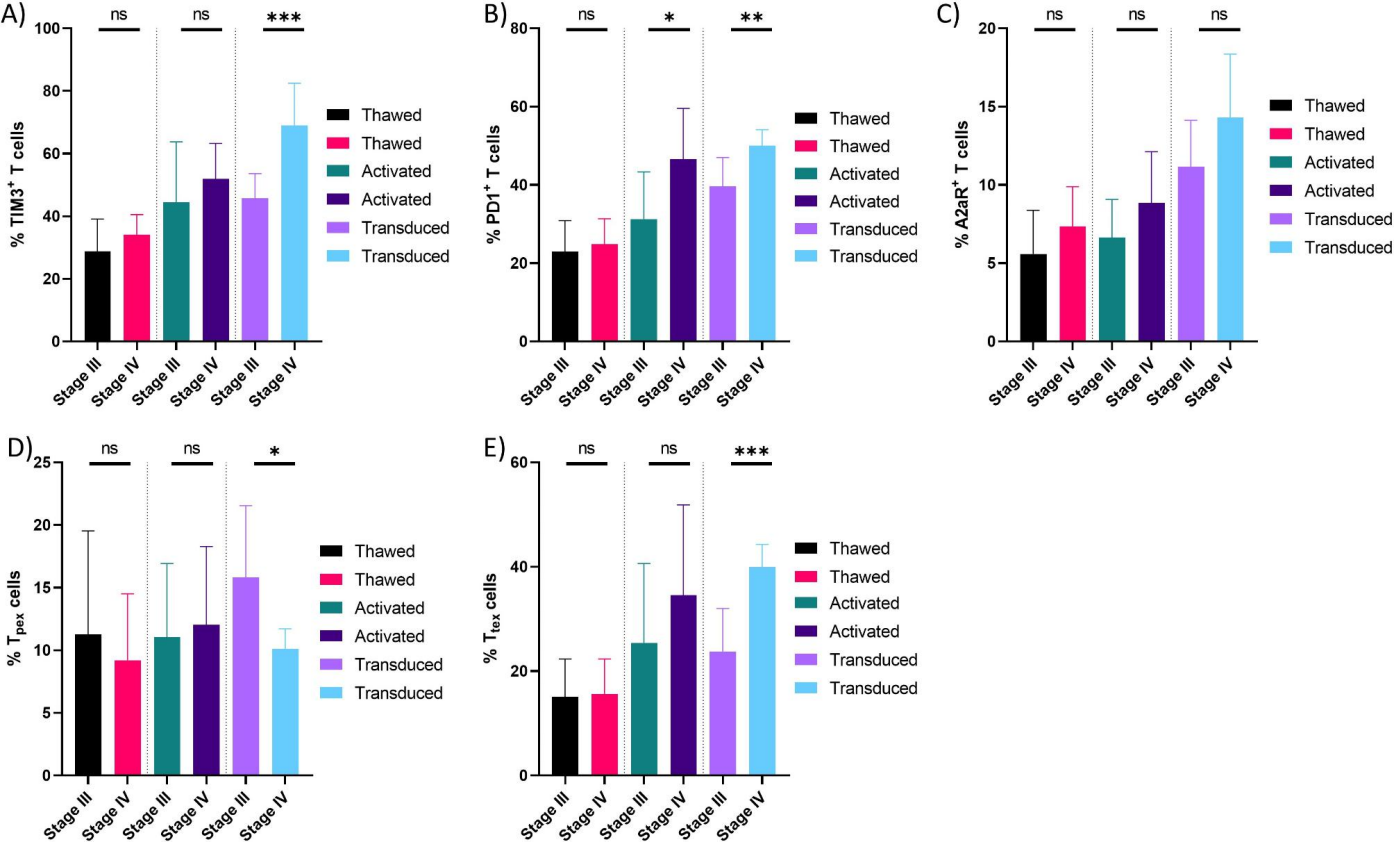
Differential expression of immune inhibitory receptors in different cancer stages during CAR T cell manufacturing. Cell surface expression levels of TIM3 **(A)**, PD1 **(B)**, A2aR **(C)**, and the frequency of T_pex_**(D)** and T_tex_**(E)** cells at different steps of CAR T cell manufacturing process for different stages of EOC. Data from stage III patients (n = 18) and stage IV patients (n = 12) were analyzed using Welch’s t-test. *P < 0.05; **P < 0.01; ***P < 0.001. Data are presented by mean ± SD.

We also investigated the effect of cancer stage on inhibitory receptor expression. Our analysis revealed that the stage of EOC (stage III vs. stage IV) can affect PD1 + and TIM3 + T cell population during CAR T cell manufacturing process, although the baseline population is equal. Specifically, mesoCAR T cells generated from T cells of stage IV had higher levels of TIM3 + mesoCAR T cells compared to CAR T cells generated from T cells of stage III EOC patients (Fig. 4A). In terms of PD1 population, PD1 + T cells were significantly upregulated in both activated T cells and mesoCAR T cells from T cells of stage IV patients compared to stage III patients (Fig. 4B). Notably, no significant difference in frequency of A2aR + cells were observed in CAR T cells between stages III and IV of EOC patients (Fig. 4C). Furthermore, our data revealed that the frequency of T_pex_ and T_tex_ cell subsets was not different in stage III and IV of EOC patients. However, upon mesoCAR T cell production, the frequency of T_pex_ cell subset was significantly lower in stage IV compared to stage III (Fig. 4D). Conversely, the frequency of T_tex_ cell subset was significantly higher in stage IV compared to stage III (Fig. 4E). These findings altogether suggest that T cells of late-stage EOC patients are more prone to exhaustion and dysfunction based on the current CAR T cell manufacturing process, which may result in inferior antitumor function in vivo.

### CarboTaxol regimen decreases in vitro clonal expansion and expression of inhibitory receptors in T cells and CAR T cells

For over 25 years, CarboTaxol has been a standard chemotherapy regimen in the treatment of advanced ovarian cancer, and other solid tumors such as cervical cancer, endometrial cancer, non-small cell lung cancer, and thymic carcinoma [22–25]. As a result, we aimed to investigate the effect of CarboTaxol treatment on the expansion of patient-derived T cells and expression of immune inhibitory receptors during the CAR T cell manufacturing process.

**Fig. 5:**
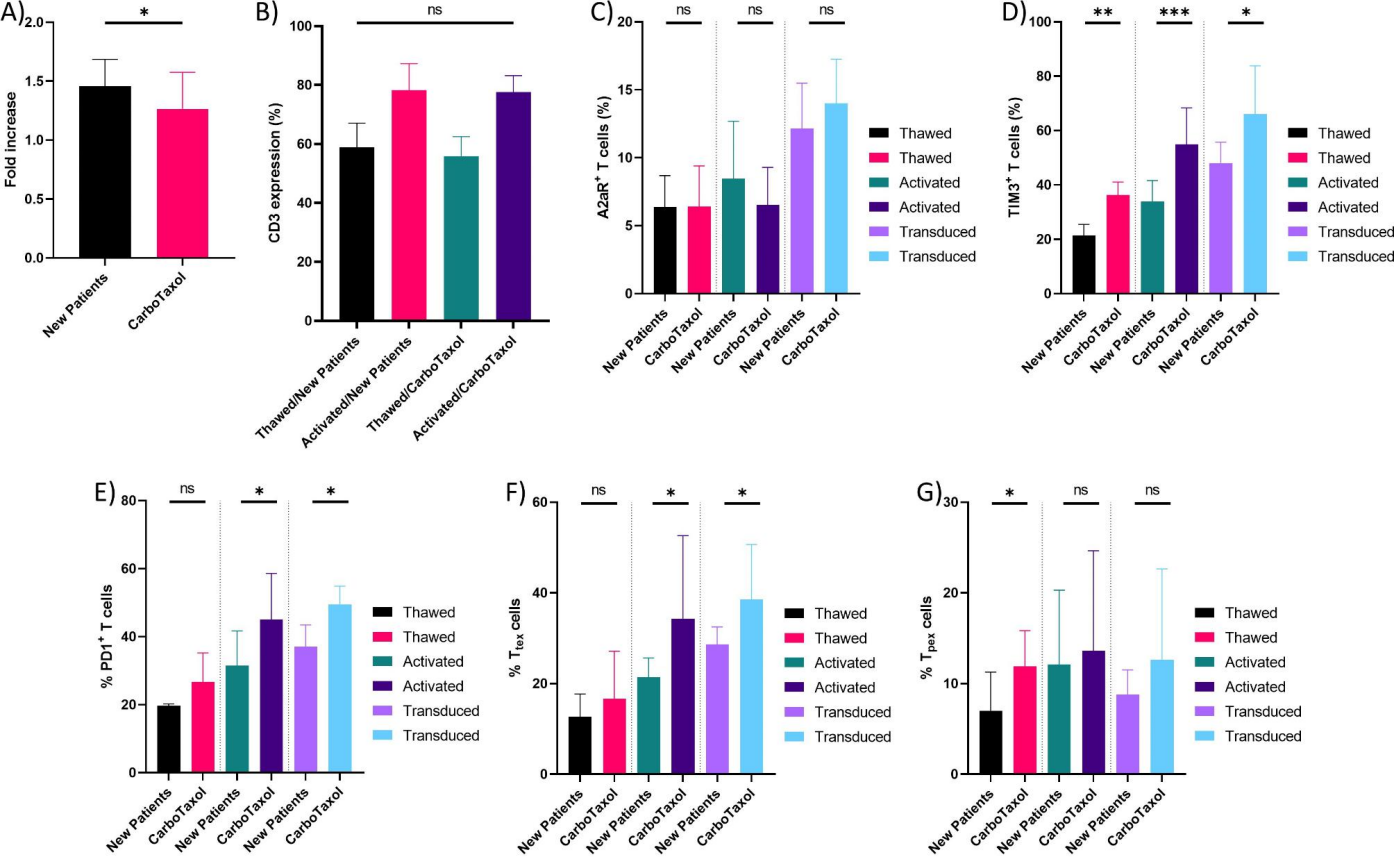
Effect of CarboTaxol treatment on T cell expansion, frequency, and expression pattern of immune inhibitory receptors during CAR T cell manufacturing process. Fold increase of T cells from CarboTaxol-treated and untreated patients post T cell activation **(A)**. CD3 expression after T cell activation in CarboTaxol treated and newly diagnosed patients **(B)**. The percentage of A2aR **(C)**, TIM3 **(D)**, PD1 **(E)** positive cells, and the frequency of T_tex_**(F)** and T_pex_ cells **(G)** during CAR T cell manufacturing process in CarboTaxol-treated and newly diagnosed patients. Data from CarboTaxol-treated patients (n = 20) and new patients (n = 10) were analyzed using Welch’s t-test. *P < 0.05; **P < 0.01; ***P < 0.001. Data are presented by mean ± SD.

The PBMCs of CarboTaxol-treated and untreated patients (i.e., newly diagnosed EOC patients) were first activated. After 4 days of activation, fold expansion of T cells from treated patients compared to newly diagnosed EOC patients was moderately decreased (Fig. 5A). Additionally, the purity of T cells from newly diagnosed patients was slightly higher compared to T cells from CarboTaxol-treated patients (Fig. 5B). Next, we investigated whether there are significant differences in the T cell population in terms of expression of immune inhibitory molecules after T cell activation and transduction between these two groups. Our analysis showed a slight decrease in the population of A2aR + T cells from CarboTaxol-treated patients after activation, but this decrease was not statistically significant (Fig. 5C). Interestingly, we found a significant increase in the levels of TIM3 + and PD1 + T cell population after activation and transduction of T cells from CarboTaxol-treated patients compared to untreated patients (Fig. 5D and E). Despite the same frequency of T_tex_ cells before T cell activation, these cells were enriched in CarboTaxol-treated patients’ samples, in both activated T cells and mesoCAR T cells (Fig. 5F). On the other hand, our data revealed that freshly isolated T cells from CarboTaxol-treated patients have a higher percentage of T_pex_ cells in their peripheral blood compared to newly diagnosed patients (Fig. 5G). However, upon T cell activation and transduction, the abundance of T_pex_ cell subset was not significant in these two groups. In conclusion, it seems that mesoCAR T cells generated from CarboTaxol-treated patients’ T cells undergo intrinsic changes in the expression of immune inhibitory receptors, which are reflected by the acquisition of a more differentiated hypofunctional phenotype during CAR T cell manufacturing.

## Discussion

Nowadays, CAR T cell therapy is being used in clinics for several types of cancers. Still, its clinical efficacy must improve. Here in this study, we found that T cells taken from EOC patients are inherently impaired and express higher levels of immune inhibitory receptors compared to healthy controls, which has also been observed in other cancers [26–28]. Next, we observed that CAR T cell manufacturing process promotes the frequency of T cells positive for specific immunoinhibitory receptors which are responsible for CAR T cell exhaustion and dysfunction. Importantly, we have previously shown that signaling through A2aR and TIM3 receptors can hinder the effector function of mesoCAR T cells generated from healthy donors’ T cells [29–31]. Therefore, genetic and/or pharmacological targeting of immune inhibitory receptors before and/or during CAR T cell production or even after infusion through combinatorial blockade of multiple immune inhibitory receptors in the patients with elevated level of these receptors may enhance the clinical efficacy of CAR T cell therapy [30–33]. Additionally, during the manufacturing process of CAR T cells, T cell activation can lead to expansion of T_tex_ cell subset. Thus, modifying culture conditions or implementing modified protocols that utilize non-activated T cells may be beneficial in generating less differentiated, highly cytotoxic CAR T cells [34].

CAR T cell therapy is often used as the last line of treatment in cancer patients who previously received various radio- and/or chemo-therapeutic regimens. These therapeutic regimens may affect the T cell functionality. In our study, we observed that EOC patients who received conventional treatments (CarboTaxol) had higher frequency of potentially scarred T cells (T cells positive for A2aR, TIM3, or PD1) in manufactured CAR T cells compared to newly diagnosed patients. This finding suggests that CAR T cell therapy as a last line of treatment in EOC patients may minimize the expected clinical outcome. Interestingly, we also found that clonal expansion of T cells is somehow impaired in CarboTaxol-treated patients, although previous findings have demonstrated an enhanced proliferation capability of antigen-specific tumor-reactive T cells following CarboTaxol treatment [35]. Moreover, we observed that the frequency of T_pex_ cell subset is significantly higher in CarboTaxol-treated patients indicating that these patients might be more responsive to PD1 therapy. Furthermore, we showed that the cancer stage can influence the frequency of both progenitor and terminally exhausted CAR T cell subsets. Additionally, we noted that following CAR T cell production, the frequency of T_pex_ cells is declined (Fig. 4D), especially in late-stage patients, while the frequency of T_tex_ cells is increased (Fig. 4E). This may further result in a decrease in the efficacy of combination therapies with CAR T cells and immune checkpoint inhibitor(s) in clinical practice. Therefore, combining the knowledge from prior interventions and immune-related gene signature of patients, especially EOC patients, may potentially predict clinical outcome of therapy.

Considering our results, utilizing PBMCs of healthy donors in preclinical studies to determine CAR T cell antitumor efficacy could be misleading due to the increased expression of inhibitory receptors in PBMCs of cancer patients. Further investigations are needed to assess the cytotoxic function and proliferation of CAR T cells produced from cancer patients compared to healthy individuals. Furthermore, we only characterized T cells from EOC patients in our experiments and further studies are required to determine if T cells from other types of cancer show similar results. Therefore, it seems that identification of intrinsic T cell dysregulation(s) through next-generation technologies and correcting these dysregulation(s) either through metabolic reprogramming, epigenetic reprogramming, utilizing gene silencing/gene-editing technologies or gene overexpression strategies, before or during CAR T cell production, may potentially overcome these intrinsic dysregulation(s) of patient-derived T cells and further enhance therapeutic efficacy of CAR T cells in solid tumors.

## Conclusion

Altogether, the current study demonstrates that population of TIM3+, A2aR + and PD1 + cells are upregulated before, and during different phases of CAR T cell manufacturing; highlighting the importance of revisiting the current CAR T cell production protocols, perhaps by development of innovative protocols which do not require pre-activation of T cells. Considering intrinsic and extrinsic factors affecting functional quality of T cells before, and during CAR T cell manufacturing, we believe that in future studies, it will be of interest to investigate targeting multiple inhibitory checkpoint receptors either pharmacologically or genetically to not only counteract cellular exhaustion, but also further protect CAR T cells against tumor-induced immunosuppression and thereby improve their therapeutic outcome.

## Electronic supplementary material

Below is the link to the electronic supplementary material.

Supplementary Material 1

## Data Availability

Data are available upon reasonable request from corresponding author.

## List of abbreviations

A2aR: Adenosine 2a receptor
CAR T cells: Chimeric antigen receptor T cells
mesoCAR T cells: Fully human anti mesothelin CAR T cells
PD1: Programmed cell death 1
TIM3: T-cell immunoglobulin and mucin-domain containing-3
T_pex_: Progenitor exhausted T cells
T_tex_: Terminally exhausted T cells
