# Supplementary material for: The inhibitory receptors PD1, Tim3, and A2aR are highly expressed during mesoCAR T cell manufacturing in advanced human epithelial ovarian cancer
Source: Cancer Cell Int. 2023 May 27;23:104. doi: 10.1186/s12935-023-02948-0 (PMC10225096; doi:10.1186/s12935-023-02948-0)
Supplement: Supplementary file 1 — Supplementary Material 1 [file 12935_2023_2948_MOESM1_ESM.docx]

**Table S1. Clinical and demographic data of patients with epithelial ovarian cancer.**

| **Patients** | **Age** | **Sex** | **Stage** | **Status** |
| --- | --- | --- | --- | --- |
| P1 | 44 | Female | IV | New patient |
| P2 | 51 | Female | IV | CarboTaxol |
| P3 | 59 | Female | III | CarboTaxol |
| P4 | 43 | Female | III | CarboTaxol |
| P5 | 65 | Female | IV | New patient |
| P6 | 57 | Female | III | CarboTaxol |
| P7 | 55 | Female | III | New patient |
| P8 | 52 | Female | III | CarboTaxol |
| P9 | 61 | Female | IV | CarboTaxol |
| P10 | 47 | Female | IV | CarboTaxol |
| P11 | 59 | Female | III | New patient |
| P12 | 42 | Female | III | CarboTaxol |
| P13 | 39 | Female | III | CarboTaxol |
| P14 | 47 | Female | III | CarboTaxol |
| P15 | 62 | Female | IV | CarboTaxol |
| P16 | 53 | Female | III | CarboTaxol |
| P17 | 49 | Female | IV | New patient |
| P18 | 55 | Female | IV | CarboTaxol |
| P19 | 48 | Female | IV | CarboTaxol |
| P20 | 67 | Female | IV | CarboTaxol |
| P21 | 44 | Female | III | New patient |
| P22 | 56 | Female | III | CarboTaxol |
| P23 | 58 | Female | IV | New patient |
| P24 | 43 | Female | III | CarboTaxol |
| P25 | 52 | Female | III | New patient |
| P26 | 64 | Female | III | New patient |
| P27 | 61 | Female | III | CarboTaxol |
| P28 | 38 | Female | III | CarboTaxol |
| P29 | 52 | Female | IV | New patient |
| P30 | 58 | Female | III | CarboTaxol |


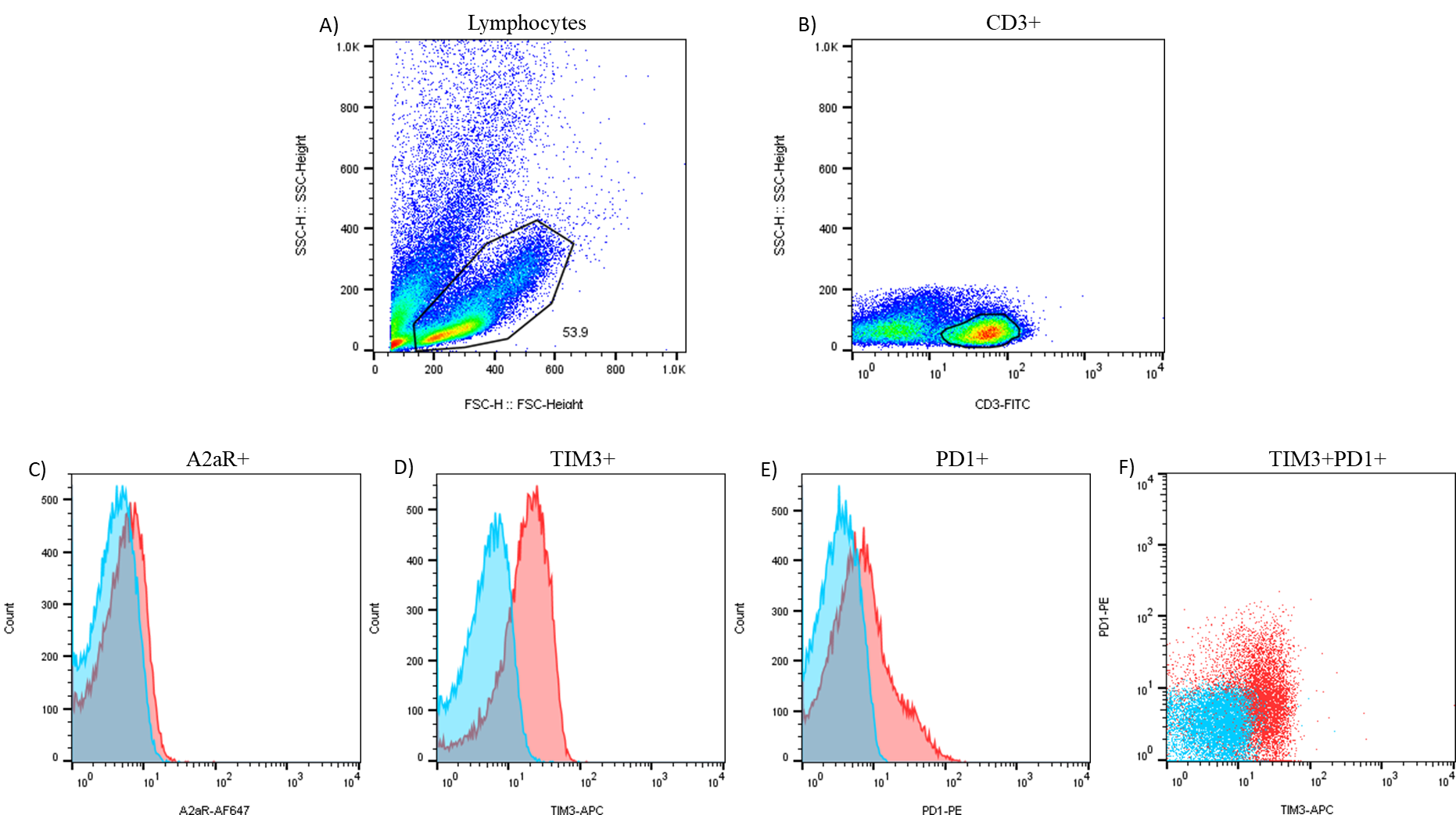


**Figure S1. Gating strategy for various CD3+ T cell subsets based on cell surface expression of TIM3, PD1 and A2aR.** Lymphocytes were gated based on size and granularity (A). T cells were then gated using SSC-H vs. CD3-FITC channel for further analysis (B). The expression levels of A2aR, TIM3, and PD1 in CD3+ gated cells are represented in histograms (C, D, E). The frequency of T_pex_ and T_tex_ cells were determined using a two-parameter density plot (F).


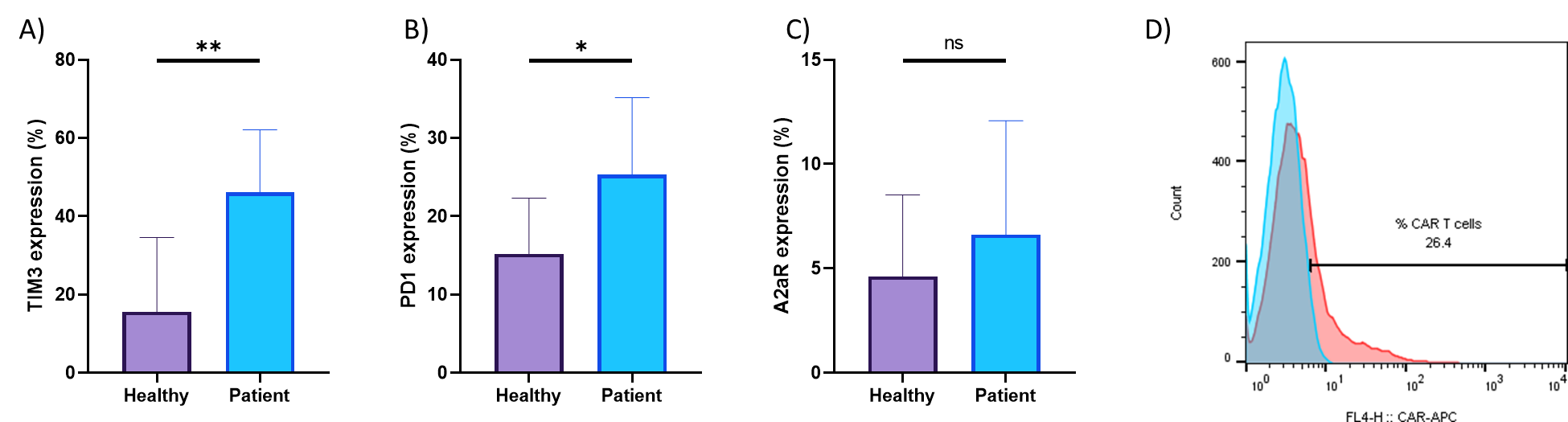


**Figure S2.** Expression levels of TIM3 (A), PD1 (B), and A2aR (C) in PBMCs of patients and healthy controls after thawing. Representative overlay histogram showing the CAR expression level post transduction (D). Data from healthy donors (n=30) and EOC patients (n=30) were analyzed using Welch’s t-test (A, B, and C). **P* < 0.05; ***P* < 0.01. Data are presented as mean ± SD.


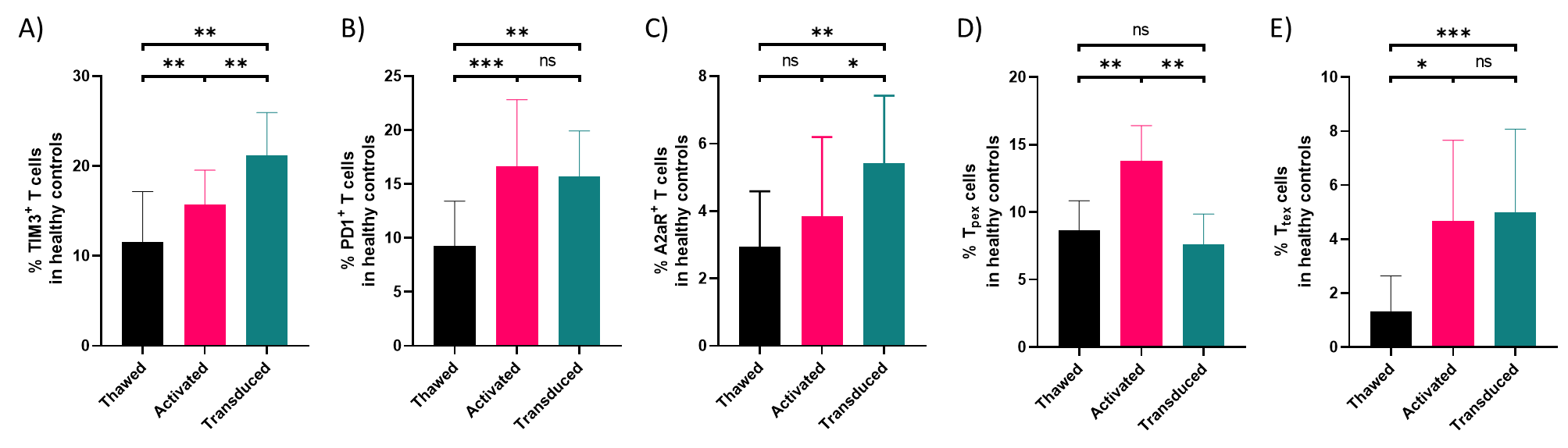


**Figure S3. The expression pattern of inhibitory receptors during CAR T manufacturing (healthy donors).** Expression pattern of TIM3 (A), PD1 (B), and A2aR (C) in healthy T cells during CAR T cell manufacturing steps. The frequency of T_pex_ (D) and T_tex_ (E) cells during manufacturing of CAR T cells in T cells of healthy controls. Data from healthy donors (n=30) were analyzed using RM one-way ANOVA and Tukey multiple comparison test. **P* < 0.05; ***P* < 0.01; ****P* < 0.001. Data are presented as mean ± SD.
